# Gene Expression Profiles in Relation to Tension and Dissociation in Borderline Personality Disorder

**DOI:** 10.1371/journal.pone.0070787

**Published:** 2013-08-12

**Authors:** Christian Schmahl, Lars Arvastson, Joseph A. Tamm, Martin Bohus, Aicha Abdourahman, Irina Antonijevic

**Affiliations:** 1 Department of Psychosomatic Medicine and Psychotherapy, Central Institute of Mental Health, Medical Faculty Mannheim/Heidelberg University, Mannheim, Germany; 2 H. Lundbeck A/S, Valby, Denmark; 3 Lundbeck Research USA, Paramus, New Jersey, United States of America; BioScience Project, United States of America

## Abstract

The biological underpinnings of borderline personality disorder (BPD) and its psychopathology including states of aversive tension and dissociation is poorly understood. Our goal was to examine transcriptional changes associated with states of tension or dissociation within individual patients in a pilot study. Dissociation is not only a critical symptom of BPD but has also been associated with higher risk for self-mutilation and depression. We conducted a whole blood gene expression profile analysis using quantitative PCR in 31 female inpatients with BPD. For each individual, two samples were drawn during a state of high tension and dissociation, while two samples were drawn at non-tension states. There was no association between gene expression and tension states. However, we could show that Interleukin-6 was positively correlated to dissociation scores, whereas Guanine nucleotide-binding protein G(s) subunit alpha isoforms, Mitogen-activated protein kinase 3 and 8, Guanine nucleotide-binding protein G(i) subunit alpha-2, Beta-arrestin-1 and 2, and Cyclic AMP-responsive element-binding protein were negatively correlated to dissociation. Our data point to a potential association of dissociation levels with the expression of genes involved in immune system regulation as well as cellular signalling/second-messenger systems. Major limitations of the study are the the possibly heterogeneous cell proportions in whole blood and the heterogeneous medication.

## Introduction

Borderline Personality Disorder (BPD) affects about 3% of the general population [1] and is closely related to traumatic life experiences [2] and displays a high comorbidity with depressive illness [3]. Instability in interpersonal relationships and self-image as well as emotion dysregulation are core symptoms of BPD (American Psychiatric Association, 1994). Emotion dysregulation and traumatic remembrance are often accompanied by the experience of dissociation, which can be defined as an altered state of consciousness causing impairments in body awareness, perception, and memory. Dissociative states are very prevalent in BPD [4], [5] and are often triggered by emotional arousal or aversive tension [6], [7]. Dissociation in BPD has been associated with higher risk for self-mutilation and depression, also higher rate of reported childhood abuse was observed among patients who dissociated [8].

There is growing concern with currently available phenotypic tools for the assessment of BPD psychopathology. Therefore, our exploratory study aimed to identify objective blood marker profiles associated with specific disease states and to examine the use of such markers to improve our understanding of the underlying biology. We chose to study gene expression profiles since standardized processes exist that permit easy collection and quick and reliable preservation of gene transcripts in blood. We focused on genes that have been linked to depressive symptoms, given the more abundant literature and independent replication of these findings, and among these we preferentially focused on intracellular signalling factors, because a good correlation between gene and protein expression has been shown [9], [10]. We hypothesized that for a subset of the genes in our panel transcriptional differences could be related to the state of tension and dissociation in patients with BPD.

Psychiatric disorders such as BPD are associated with (neuro-) biological alterations, which affect the brain as well as the body [11]. For example, one of the most thoroughly examined biological alterations in psychiatric disorders such as depression or posttraumatic stress disorder (PTSD) is the dysregulation of the hypothalamic-pituitary-adrenal (HPA) axis, which is likely to contribute to systemic manifestations of depressive illness and PTSD [12]. In addition, immune alterations and activation of the inflammatory response system can accompany depression [13]–[15]. One of the best studied cytokines in depression is interleukin (IL)-6 for which increased concentrations could be confirmed meta-analytically [14], [15]. Plasma-IL6-levels covaried negatively with hippocampal volumes [16], which is interesting in the context of reduced hippocampal volume being robustly associated with BPD [17]–[19]. In the CSF, high levels of IL-6 in patients with depression vs controls were reported with highest elevations in depressed patients with a recent violent suicide attempt [20]. In patients with BPD, immune dysregulation has not been as widely studied, but. increased IL-6 levels were found in the plasma of BPD patients [21], and IL-1ß levels increased significantly stronger after a glucose challenge in BPD patients as compared to healthy controls [22].

Besides cytokines, transcriptional factors have been reported to show alterations in mental disorders [23]–[25]. Cyclic AMP response element-binding protein (CREB) is a transcriptional target of the calcium signalling cascade. CREB has been reported to be a molecular marker for the response to antidepressants in neurons [26] and in patients with severe major depression [24]. Alterations of calcium signalling in the peripheral leukocytes have been reported in major depression [27]–[29]. PTSD patients had statistically lower levels of total CREB protein in lympho-monocytes than healthy control subjects [30].

Besides transcriptional factors, changes in other intra-cellular signalling pathways have been associated with multiple mental disorders. Extracellular signal-regulated kinases (ERKs) are a subfamily of the mitogen-activated protein kinases that are activated in neurons by a variety of extracellular signals, including various neurotransmitters [31]. Decreased mRNA and protein levels of ERK-1 and -2 as well as of upstream molecules of the ERK signalling cascade were found in the hippocampus and prefrontal cortex of suicide victims [32], [33]. The ERK MAP signalling pathways have been related to learning and memory as well as to PTSD psychopathology [34]. Another protein family involved in intra-cellular signalling, the ß-Arrestins, play an important role in G protein-coupled receptor desensitization. Mice chronically treated with corticosterone as a model of depressive states revealed reduced ß-Arrestin expression [9]. Furthermore, ß-Arrestin-1 levels were found to be elevated by antidepressants in rat cortex and hippocampus and reduced in leukocytes of depressed patients [35].

Interestingly, there is growing evidence that early adverse life experiences can modulate HPA activity and may potentiate abnormalities observed during an acute depressive episode [36]. In a similar vein, childhood abuse has been shown to increase secretion of IL-6 (but not other immune parameters) in subjects experiencing stressful situations later on in life [37].

Such differences in life histories may not only affect the likelihood to develop a depressive disorder, but may also influence other trauma-related pathophysiology. One such study has described a specific profile of messenger RNA expression in peripheral blood mononuclear cells in subjects who experienced a trauma and later developed posttraumatic stress disorder [38]. Specifically, the authors showed that subjects who developed PTSD had elevated expression of genes indicating immune activation, while expression of a number of genes associated with transcription and intra-cellular signalling was decreased. Another recent study showed the influence of childhood maltreatment on the methylation extent of the BDNF gene [39], opening up the hypothesis that further genes could be affected by such epigenetic changes. Gene expression studies in psychiatry have often focused on differentiating patients from control subjects. However, expression may not only vary inter-individually, but also in relation to different disease states within the same individual. Such changes may be particularly relevant to measure treatment response within a given individual, and is less prone to potential confounds across individuals such as prior life experience. Transcriptional changes can occur within minutes to hours and thus could help understand the biology of behavioral and emotional changes.

Therefore, in the present study, we used a panel of 29 genes, selected based on their association with depressive states in humans. We elected to measure gene expression profiles in patients with BPD as they display high levels of fluctuations in affective states as well as in dissociation. Our patients participated in a standardized residential intensive psychotherapy program. Thus, life circumstances were kept more constant and controlled than is the case when studying outpatients.

## Materials and Methods

### Participants

31 female patients with Borderline Personality Disorder were included in this study. All patients were inpatients at the Department of Psychosomatic Medicine and Psychotherapy, Central Institute of Mental Health Mannheim, Germany. Diagnostic procedures were conducted by trained raters and comprised the International Personality Disorder Examination (IPDE [40], inter-rater reliability: κ = .77), and the Structured Clinical Interview for DSM-IV Axis-I (SCID-I [41], κ = .69). We excluded patients with alcohol or substance abuse within the past three months. Most patients were on psychotropic medication, which mainly consisted of SSRI, atypical neuroleptics and mood stabilizers. No patient was on benzodiazepines. An attempt was made to keep patients on constant medication throughout the study, however in 16 patients changes of medication with either adding or removing prescribed drug, increases or decreases of dosage were necessary. Table S1 lists medication for all participants at the tension and the non-tension state. We exluded patients with significant medical conditions, in particular those with immunological conditions.

Mean age was 27.47±7.95, mean weight 76.22±21.73 kg. 20 out of 31 patients were smokers. Comorbid diagnoses were as follows: MDD, current depressive episode (n = 16), MDD, currently remitted (4), Dysthymia (3), past alcohol abuse (5), past lcohol dependence (2), past sedatives abuse (3), past sedatives dependence (1), past cannabinoids abuse (1), past stimulants abuse (1), past multiple substance abuse (1), Panic disorder without agoraphobia (1), Panic disorder with agoraphobia (2), Social phobia (7), Specific phobia (1), Obsessive-compulsive disorder (2), Posttraumatic stress disorder (6), Anorexia nervosa (4), Bulimia nervosa (4), Eating Disorder NOS (1), Attention-deficit hyperactivity disorder (2).

### Ethics statement

The study was conducted in accordance with the Declaration of Helsinki and was approved by the Ethics committee of the University of Heidelberg. After explanation of the procedure, written informed consent was obtained. All participants had the capacity to consent, which was checked by a psychiatrist (C.S.).

### Procedure

Blood samples for gene expression profiles were collected at two different states, a tension and a non-tension state, with one or two blood draws for each state, respectively. Blood was collected into PAXgene^TM^ blood RNA tubes, mixed by inversion several times, left at room temperature for 30 – 120 minutes, frozen at −20°C, and then transferred to −80°C for long term storage. RNA integrity is maintained for at least 5 years under such conditions.

For the tension state, patients were asked to signal to the research assistant during their stay in the hospital when they subjectively felt a high level of aversive inner tension. Level of tension was verified by assessment with the DSS (see below). The blood draws for the non-tension state were conducted approximately at the same time of the day (+/− 1 hour) as the tension blood draws. Tension Blood draws preceded non-tension blood draws in all cases. We included 91 observations from 31 subjects into the final analysis, as not in all participants, tension and non-tension stations could be sampled twice. One subject had no tension observation, nine subjects had one tension observation, and 21 had two tension observations. Nine subjects had no non-tension observation, one subject had one non-tension observation, and 21 had two non-tension observations. The study lasted for up to ten weeks.

### Gene selection

A list of 29 transcripts was selected, based on multiple criteria including reported association with depressive disorders, assumed role in depressive pathophysiology, and technical feasibility (Table S2). Using stringent selection criteria from a methodological and technical point of view, we believe that changes in the selected genes could be indicative of a pathophysiological relevance, including changes occurring in the CNS [42].

### Total RNA isolation

Extraction of RNA from PAXgene tubes was performed using the PAXgene Blood RNA MDx kit (#762431) run on a Qiagen Biorobot Universal instrument according to the manufacturer's protocol. To ensure that the RNA samples were free of contaminating genomic DNA, a second DNase digestion was added to the standard Qiagen protocol. In our experience, this reduces the level of DNA in the samples such that it accounts for less than 0.5% of the signal generated by the downstream qPCR analysis. As such, it is unnecessary to design the qPCR primers used for gene expression profiling to span intron//exon junctions. After isolation, an aliquot of the RNA was analyzed by capillary electrophoresis on a Caliper GX Lab Chip instrument to assess quality and quantity, The RNA concentrations derived from this analysis were used to normalize the RNA r to a final concentration of 13 ng/ul by the addition of water. The samples were stored at −80°C.

### cDNA synthesis and qPCR assays

Reverse transcription of 0.5 ug of total RNA into cDNA was accomplished using Superscript II (Life Technologies, # 18064–014) according to the manufacturer's protocol. Following the reaction, the cDNA was desalted using a Multiscreen PCR96 filter plate (Millipore # LSKMPCR10) and the cDNA eluted in 100 ul of water. The cDNA was quantified using the Quant-IT oligreen spectrophotometric assay (Quant-It oligreen ssDNA reagent, Life Technologies #07582). Following quantitation, all cDNA samples were normalized to a concentration of 2.5 ng/ul by the addition of water and stored at −20°C. Quantitative polymerase-chain reaction (qPCR) is preferred over whole genome array as it reliably quantifies transcription changes of small but physiologically relevant magnitude. This is in line with other recent work showing that small but quantitative changes can be used to better characterize psychiatric patient populations [43]. For qPCR, 0.5 ng of cDNA was assayed in a 5 ul reaction volume on an Applied Biosystems 7900HT Fast Real Time PCR instrument using Taqman style primers and probes (Table S3). Because the expression level of IL-6 and SERT are relatively low, the qPCR assays were conducted with 5 ng of cDNA per well to increase the signal. The qPCR master mix was BrilliantII FAST (Agilent # 600845) using the reaction conditions, cycling times and temperatures recommended by the manufacturer. Duplicate plates were run for each assay. Cycle thresholds were set manually for each run and the raw Ct values from the duplicate plates were averaged for analysis. Each assay plate also contained negative control wells (water) and 4 wells of cDNA derived from RNA extracted and pooled from the blood of 10 healthy control individuals. This served as the reference cDNA (see below).

### Normalization of gene expression and copy number determination

Averaged expression values from the seven normalization transcripts (Table S4) were used to normalize the raw gene expression data for each well. The seven normalization transcripts were selected based on their stable expression pattern in human blood as determined by the Genorm software program. The stability testing included healthy controls, PTSD patients, depressed patients, and depressed patients receiving treatment with duloxetine or escitalopram, indicating that the normalization process is not impacted by these medications. The relative gene expression in each well, including the reference cDNA, was determined by subtracting the average Ct for the transcript of interest from the average Ct of the seven normalization transcripts (the 2^−delta Ct^ method). Next, setting the relative expression level of the reference cDNA at 100%, all other samples were expressed as a percentage of the reference. Finally, these percentages were converted to copies per ng of cDNA by multiplying this percentage by the number of copies of each transcript contained in the reference cDNA. The copy number determinations for the reference cDNA were derived separately by comparing the expression level in the reference to a standard curve of synthetic DNA for each transcript of interest.

### Psychometric measures

Immediately after each blood draw, patients filled in a scale measuring aversive tension and dissociation (Dissoziations-Spannungs-Skala, DSS [44]. This scale has 21 items assessing different aspects of dissociation (e.g. depersonalisation, derealisation, and reduced sensory perception) as well as one item for the assessment of aversive inner tension (tension item). Each week during the course of the study, patients filled in the Borderline-Symptom-List (BSL, [45]) to assess the overall severity of BPD pathology.

### Data analysis

The purpose of the data analysis was to establish links between the biology as described by the gene expression levels and the severity of BPD and the severity of tension and dissociation states. This can basically be done in two different ways. One approach is to calculate a predictive marker that given expression levels predict symptom severity. The alternative is to calculate how the expression levels will change due to symptom severity. The two approaches are similar but the first method might identify only the genes with the strongest association with disease state. The latter method will identify all genes with a significant association with the disease. Here the ambition was to understand the biology and therefore the latter approach was prefered. Hence, the expression levels were used as response variables and the symptom scores as explanatory variables in the analysis. With this approach we explicitly calculated what genes/biological processes were associated with the disease states.

BSL and DSS scores represent different time resolution of the disease progression. The overall progression is represented by the BSL and the acute state is represented using the DSS. The BSL score taken once weekly does not necessarily coincide in time with the occasions where blood and BSL scores were collected. The nearest BSL rating before the blood sampling occasion was assigned to the observation representing the long term progression of the disorder.

Four variables describing the disease status were of potential interest. The quantitative BSL score, the DSS total score, the DSS tension sub-score and a categoricalindicator for tension/non-tension state. Data are limited and the explanatory variables are correlated and describe different aspects of the same disorder. We analysed the association between the variables and expression level one by one. Each approach involved one mixed effect model for each gene with expression level as dependent variable.

One of the disease markers plus the subject identity was used as explanatory variables in each approach. We analyzed the following groups of explanatory variables for each gene: (1) subject identities and BSL score, (2) subject identities and tension/non-tension state, (3) subject identities and DSS tension item score, (4) Subject identities and DSS total score. Mixed effect models were chosen for the analysis to account for within-subject correlation between measurements. The subject identities were incorporated as a random effect and all other variables were treated as fixed factors. In order to compensate for the non-symmetric distribution of the gene expressions levels, logarithmic expression values were chosen. The analysis was performed using “proc mixed” in SAS version 9.2. Four mixed effect models were built in this way. The only difference between them was the choice of disease score used as explanatory variable. All 91 non-missing observations from the 31 subjects went into the model. No imputation of missing data was performed.

The number of genes is similar to the number of patients studied. Repeating the analysis of significant regression coefficients for 29 genes will overestimate the number of genes associated with BPD. One traditional approach avoiding this is using Bonferroni correction for multiple testing which will give a shorter list of significantly associated genes. At a specified level of significance all associated genes on the list will be correct findings. Alternatives exist that will allow a few false findings among the associated genes. The benefit is an increased sensitivity to genes associated with BPD. To identify groups of genes that interacts with BPD we chose to use false discover rate, FDR, as selection criteria. The actual calculations follow the Benjamini-Hochberg procedure for false discovery rates. We were ready to accept genes with a false discovery rate below 20% due to the exploratory nature of the study. Among the genes identified at a FDR of 0.20 there is expected to be 20% of false findings. Hence, by using false discovery rate as a criterion we are accepting to have some errors in the list of affected genes instead of potentially not finding a significant group of affected genes using the more conservative Bonferroni correction method.

## Results

### DSS total score and tension score

**Figure 1:**
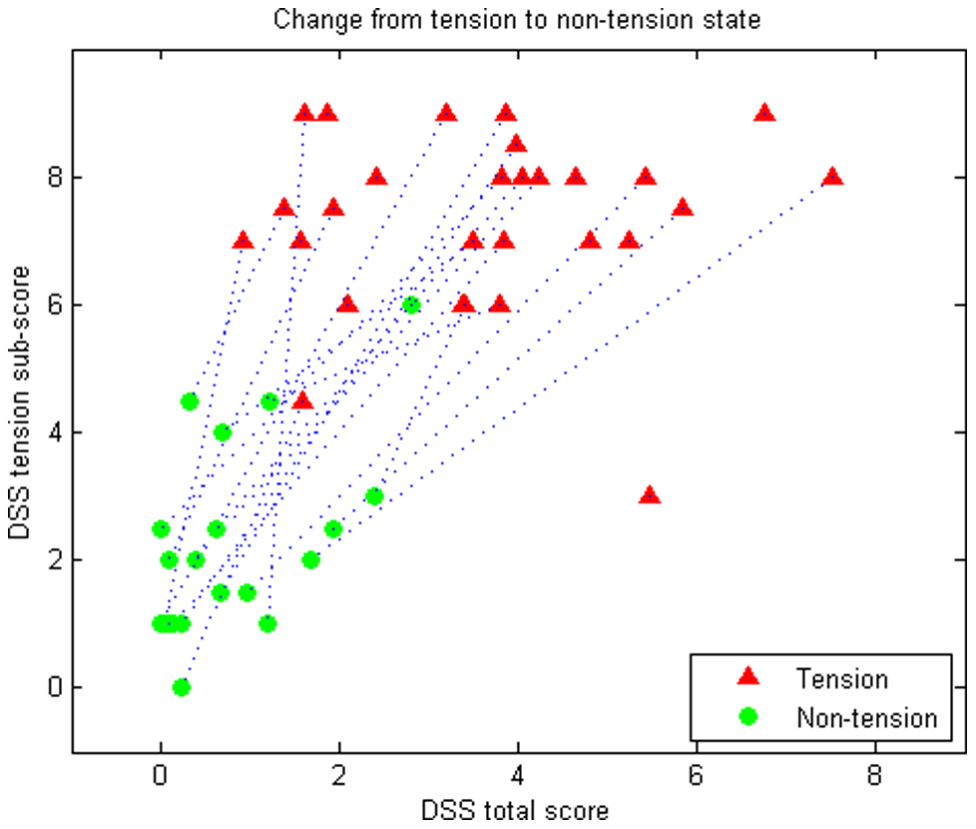
DSS total scores and tension sub-scores for tension and non-tension state. Scores are averages over the two observations in each state. The dashed line represents the patients and the two states of each patient.

Figure 1 shows distribution of DSS total scores and tension scores. A better separation between groups was found for tension scores (y-axis) than for total scores (x-axis). Hence, the tension score was assumed to be better at capturing the state than the total score.

### Gene expression analyses

No significant effects were found at gene expression level for BSL score, Tension/Non-tension state, and DSS tension item at a FDR of 20% (see Tables S5, S6, and S7). However, 14 genes showed a significant correlation with DSS total score at a FDR of 20%.

Guanine nucleotide-binding protein G(s) subunit alpha isoforms short (GNAS), Interleukin-6 (IL6), Interleukin-1B (IL1B),Mitogen-activated protein kinases 1,3, and 8 (MAPK1, MAPK3, MAPK8), Guanine nucleotide-binding proten G(i) subunit alpha-2 (GNAI2), Beta-arrestin-1 (ARRB1), Beta-arrestin-2 (ARRB2), Cyclic AMP-responsive element-binding protein (CREB1), Glucocorticoid receptor (NR3C1), Regulator of G-protein signaling 2 (RGS2), Synaptic vesicular amine transporter (SLC18A24), and Prolylendopeptidase (PREP) (see Table 1). All of these genes with the exception of IL-6 showed a negative correlation, i.e. reduced expression with increasing DSS total scores, while IL-6 expression was higher with increasing DSS total scores.

**Table 1:** Gene expression level associated with DSS total score sorted by FDR.

Gene	Estimated regression coefficients	FDR	p-value
GNAS	−0.05223	0.11065	0.0103
IL6	0.05219	0.11065	0.0119
MAPK3	−0.04340	0.11065	0.0135
GNAI2	−0.04864	0.11065	0.0153
ARRB2	−0.05073	0.11439	0.0242
CREB1	−0.03976	0.11439	0.0289
ARRB1	−0.03937	0.11439	0.0307
MAPK8	−0.03770	0.11439	0.0316
IL1B	−0.04611	0.14406	0.0480
NR3C1	−0.03488	0.14406	0.0506
RGS2	−0.04235	0.14406	0.0546
SLC18A2	−0.03917	0.15303	0.0633
PREP	−0.02603	0.16873	0.0756
MAPK1	−0.02939	0.19587	0.0946
ODC1	−0.03759	0.20242	0.1047
SLC6A4	−0.04805	0.26513	0.1463
S100A10	−0.01472	0.42987	0.2520
MAPK14	−0.02145	0.45085	0.2798
CD8A	0.02818	0.47601	0.3120
DPP4	−0.01444	0.47601	0.3283
NR3C2	−0.01975	0.59230	0.4312
DUSP1	−0.02013	0.59230	0.4493
ADA	0.01993	0.60730	0.4817
P2RX7	−0.01231	0.66572	0.5509
IDO1	−0.01393	0.74882	0.6455
CD8B	0.005458	0.79109	0.7209
TSPO	−0.01115	0.79109	0.7365
IL8	−0.00725	0.81301	0.7850
ATF2	0.002497	0.82935	0.8294

## Discussion

To our knowledge, this is the first study investigating longitudinal intra-individual gene expression profiles in blood in patients with borderline personality disorder. We recognize that this is a small study and thus needs replication of the data in a larger study. On the other hand, our design to compare longitudinal intraindividual changes in gene expression during different emotional states in BPD is in line with the suggestion that within-subject comparisons may provide better insights into the pathophysiology than cross-sectional comparisons [23]–[25]. One recent study reported intraindividual comparisons in BPD found that changes in BDNF methylation were influenced by symptoms of depression [39]. This finding together with an earlier study showing that dissociation in BPD is associated with higher risk for depression [8] are in line with our findings of an association between transcriptional changes of genes linked to depressive symptoms and dissociation scores in BDP.

We intraindividually compared patients in two different states of the disease and analyzed gene expression in relation to acute states, in this case tension and dissociation. With a relatively liberal statistical threshold due to the exploratory nature of our study, we found no effect of acute tension or overall symptom severity on gene expression profiles. However, the expression of several genes, including those involved in immune system activation, HPA axis regulation as well as intracellular signalling/second-messenger systems were associated with the level of dissociation, one of the most prominent psychopathological features of the disorder and closely related to states of tension. While the genes were selected mostly based on their association with depressive states, the shared symptomatology between depressive states and emotional disturbances in borderline personality disorders and the risk for depression in patients with greater dissociation could point to shared biological underpinnings. This is in line with the notion that biological research into psychiatric disorders may have to cross diagnostic boundaries to advance our understanding of the pathophysiology of complex clinical presentations [46]. Thus the selected genes and the reported changes may be of relevance beyond BPD. Our data also add to the growing literature on relevant correlations between protein and gene expression for factors involved in intracellular processes [9], [10] as well as the relevance of blood markers for CNS disorders [42], [47].

Dissociation is characterized by a disintegration of perception, consciousness, identity, and memory (DSM-IV-TR). Dissociative symptoms are part of the diagnostic criteria in BPD and several studies revealed frequent dissociative states in BPD [5], [8], [48]. Stiglmayr and coworkers [6] demonstrated dissociative states to be significantly associated with every-day distress in patients with BPD. The neurobiological underpinnings of dissociation are mostly unknown. Neuroimaging studies point to decreased activity in the amygdala in conjunction with increased medial prefrontal/anterior cingulate activity [49], [50].

The genetic background of dissociation is also an area which lacks sufficient research. Twin studies revealed a significant influence of genetic factors on the phenotypic expression of dissociation [51] [52]. Koenen and colleagues [53] found that genetic polymorphisms of FKBP5, which is involved in glucocorticoid receptor regulation, explains up to 14% of the variance in dissociation of children during and after accidents. BDNF polymorphisms were associated with lower dissociation scores [54]; this study also revealed a gene-environment interaction between the Catechol-O-methyltransferase (COMT) val158met-polymorphism and traumatic life events: For the val/val-genotype, greater traumatic experience was associated with increasing dissociation scores, while the met/met-genotype showed an opposite picture. For the serotonin transporter gene, an interaction of the short variant and traumatic neglect was found in the generation of dissociative symptoms [55].

There have been several investigations of the HPA axis in BPD. Dexamethasone suppression has been found to be neither particularly sensitive nor specific for BPD [56]–[58]. Lieb and coworkers [59] collected salivary cortisol during every-day life conditions in patients with BPD and found significantly higher salivary cortisol levels than in healthy controls. Nater and coworkers [60] reported cortisol hyporeactivity to a psycho-social stressor. Taken together, these data suggest that in BPD blood markers may be changed under both stable as well as stress conditions. In contrast to the HPA axis, immune dysregulation has not been as widely studied in BPD. One study found increased IL-6 levels [21], while IL-1ß levels increased significantly stronger after a glucose challenge in BPD patients as compared to healthy subjects [22]. In the current study, IL-6 gene expression was the only transcript to show a positive correlation with acute dissociation levels at an FDR-level of 11%. Thus, our data add to the growing literature on correlations between protein and gene expression, at least for some genes, and also provide insights into the pathophysiology of specific disease states.

Besides immune system regulation genes, we found genes involved in signalling/second-messenger systems as well as genes related to transcriptional factors to be potentially related to dissociation in BPD. Changes in expression of beta-Arrestin-1 and -2, mitogen-activated protein kinase 3 as well as extracellular signal-regulated kinase 1 were negatively correlated to dissociation scores at FDR-levels between 11 and 11.5%. It is of interest in this context that decreased ERK-1-levels were also found in the hippocampus and prefrontal cortex of suicide victims [32], [33]. IL-6 was found to be elevated in the CSF of suicide attempters [20]. The suicide rate of patients with BPD is very high with approximately 10% of all patients committing suicide [61]. Further research is needed to disentangle the interaction between dissociation and disturbances of signalling/second-messenger systems in patients with BPD and/or suicidal behavior.

Cyclic AMP response element-binding protein was also negatively related to dissociation scores. Alterations in CREB metabolism has been related to depression [24], [27]–[29]. In a recent study, PTSD patients (who also often show elevated dissociation scores) had statistically lower levels of total CREB protein than healthy control subjects [30].

Overall, our data on blood gene expression changes are in good agreement with reported protein changes in psychiatric patients with depressive symptoms. Specifically, we found a negative association between selected cellular signalling molecules and dissociation and a positive association for IL-6, similar to published protein data in depression and related disease states by showing [9], [13]–[15].

Limitations of our study include the fact that medication was not held constant in all patients and a relatively small sample size. And while many published studies on transcriptional profiles in psychiatric disorders are small and different in terms of design and gene selection and thus do not permit a direct comparison of results, medication, whenever examined, had no clear impact on the results [23], [62].

Other state-dependent symptoms besides dissociation, e.g. anxiety, which were not assessed in our study, may have confounded our results. Since in most patients, several weeks lay between the assessments of the tension and the non-tension states, other factors, e.g. subclinical infection, may have influenced the change of immune system-related gene expression. On the other hand, careful diagnostic procedures were conducted and all patients were inpatients at the same unit. Also, intraindividual comparison as in our study can be considered a way to assess emotional states and reduce confounds of other interindividual variables [23]. In addition to the intraindividual comparison, we also collected blood samples twice for each disease state, and used the average of the same disease state, thereby further reducing the impact of possible confounds. As further strength can be considered that, unlike genetics and proteomics data, the RNA infrastructure [63] may better capture also the effect of epigenetic changes that have recently been reported in psychiatric disorders, such as BPD [62], [64] .

Overall, this pilot study gave some preliminary hints at alterations in gene expression related to dissociative psychopathology in patients with BPD. Our data point to a potential association between gene expression involved in immune regulation as well as signalling/second-messenger systems and the level of dissociation in patient with BPD. Our findings, while preliminary, suggest that further research into molecular alterations in blood associated with specific disease states such as dissociation in BPD may improve our understanding of the underlying pathophysiology. In view of the shared molecular pathophysiology between psychiatric disorders such insights may be applicable to similar disease states in multiple psychiatric disorders.

## Supporting Information

Table S1 **List of medication for all participants at the tension and the non-tension state.**
(DOCX)

Table S2 **List of analyzed genes.**
(DOCX)

Table S3 **Targets and primers.**
(DOCX)

Table S4 **Normalization transcripts.**
(DOCX)

Table S5 **Gene expression level associated with BSL score sorted by FDR.**
(DOCX)

Table S6 **Gene expression level associated with Tension/Non-tension state sorted by FDR.**
(DOCX)

Table S7 **Gene expression level associated with DSS tension item sorted by FDR.**
(DOCX)
