# Supplementary material for: Gene Expression Profiles in Relation to Tension and Dissociation in Borderline Personality Disorder
Source: PLoS One. 2013 Aug 12;8(8):e70787. doi: 10.1371/journal.pone.0070787 (PMC3741306; doi:10.1371/journal.pone.0070787)
Supplement: Table S1 — List of medication for all participants at the tension and the non-tension state. (DOCX) [file pone.0070787.s001.docx]

**Table S1: List of medication for all participants at the tension and the non-tension state**

| **Pat. #** | **Medication tension state** | **Medication non-tension state** |
| --- | --- | --- |
| 1 | sertraline 75 | sertraline 75 |
| 2 | none | none |
| 3 | fluoxetine 20 | none |
| 4 | quetiapine 500, sertralinee 150, prazosin 1, esomeprazole 20, ferrosanol 1 | quetiapine 500, sertralinee 150, prazosin 4, ferrosanol 1 |
| 5 | citalopram 40, quetiapine 250, l-thyroxine 75 µg | citalopram 40, l-thyroxine 125 µg |
| 6 | venlafaxine 225, naltrexone 200, olanzapin 2.5, esomeprazole 20 | venlafaxine 225, naltrexone 200, esomeprazole 20 |
| 7 | perazine 100, venlafaxine 300, valproate 450, metoprolol 100, esomeprazole 40 | perazine 150, venlafaxine 300, valproate 450, metoprolol 100, esomeprazole 40 |
| 8 | venlafaxine 150, bupropion 450, quetiapine 700, lamotrigine 250, pregabalin 200 | venlafaxine 150, bupropion 450, quetiapine 700, lamotrigine 250, pregabalin 200 |
| 9 | duloxetine 120, pipamperone 60, pantoprazole 40, ramipril 7.5, metformin 1500, cyproterone 10, dexamethasone 0.5 | duloxetine 120, pipamperone 60, escitalopram 20, pantoprazole 40, ramipril 7.5, metformin 1500, cyproterone 10, dexamethasone 0.5 |
| 10 | loratadine 10 | none |
| 11 | none | none |
| 12 | venlafaxine 150, quetiapine 25 | venlafaxine 150, valerian 180 |
| 13 | methylphenidate 30 | methylphenidate 30 |
| 14 | berlthyrox 100 µg | none |
| 15 | venlafaxine 225, mirtazapine 15, esomeprazole 20, acetylcysteine 600 | venlafaxine 225, mirtazapine 15, esomeprazole 20, acetylcysteine 600 |
| 16 | sertraline 100, quetiapine 100 | methylphenidate 55 |
| 17 | citalopram 40, quetiapine 100, prazosin 3, diclofenac 75, pantoprazole 20, budesonide 0,4 | citalopram 40, quetiapine 150, prazosin 6, pantoprazole 40, budesonide 0,4 |
| 18 | topiramate 200, aripiprazole 2.5, escitalopram 20 | topiramate 200, aripiprazole 2.5, escitalopram 20 |
| 19 | venlafaxine 150, pregabalin225, quetiapine 200 | venlafaxine 150, pregabalin 300 |
| 20 | trimipramine 50, escitalopram 15 | trimipramine 50, escitalopram 15 |
| 21 | quetiapine 300, bisacodyl 4 | quetiapine 300, ferrosanol 200, buprenorphine 0.4 |
| 22 | none | none |
| 23 | escitalopram 10 | escitalopram 10 |
| 24 | none | none |
| 25 | citalopram 10, valproate 900, ziprasidone 80, risperidone 1.5 | ivabradine 10, citalopram 10, valproate 900, quetiapine 450 |
| 26 | mirtazapine 15, escitalopram 10 | mirtazapine 15, escitalopram 15 |
| 27 | metoprolol 50, venlafaxine 150, valproate 300 | metoprolol 50, venlafaxine 150 |
| 28 | venlafaxine 300, quetiapine 75, acamprosate 1333 | venlafaxine 300, quetiapine 75, acamprosate 1333 |
| 29 | citalopram 40, doxepine 50, quetiapine 125, prazosin 1 | citalopram 40, doxepine 25, quetiapine 125, prazosin 3 |
| 30 | quetiapine 700, venlafaxine 225, valproate 600, doxepine 75, prazosin 2, fexofenadine 360, pantoprazole 20 | quetiapine 700, venlafaxine 225, valproate 600, doxepine 75, prazosin 2, fexofenadine 360, pantoprazole 20 |
| 31 | none | none |
|  |  |  |
|  | constant medication |  |
