# Supplementary material for: Gene Expression Profiles in Relation to Tension and Dissociation in Borderline Personality Disorder
Source: PLoS One. 2013 Aug 12;8(8):e70787. doi: 10.1371/journal.pone.0070787 (PMC3741306; doi:10.1371/journal.pone.0070787)
Supplement: Table S2 — List of analyzed genes. (DOCX) [file pone.0070787.s002.docx]

**Table S 2: List of analyzed genes**

| **UniProt** | **Short ID** | **ENSMBL gene** | **UniProt recommended name** |
| --- | --- | --- | --- |
| ADA_HUMAN | ADA | ENSG00000196839 | Adenosine deaminase |
| ARRB1_HUMAN | ARRB1 | ENSG00000137486 | Beta-arrestin-1 |
| ARRB2_HUMAN | ARRB2 | ENSG00000141480 | Beta-arrestin-2 |
| CD8A_HUMAN | CD8A | ENSG00000153563 | T-cell surface glycoprotein CD8 alpha chain |
| CD8B_HUMAN | CD8B | ENSG00000172116 | T-cell surface glycoprotein CD8 beta chain |
| CREB1_HUMAN | CREB1 | ENSG00000118260 | Cyclic AMP-responsive element-binding protein |
| ATF2_HUMAN | ATF2 | ENSG00000115966 | Cyclic AMP-dependent transcription factor ATF-2 |
| DPP4_HUMAN | DPP4 | ENSG00000197635 | Dipeptidyl peptidase 4 |
| MK03_HUMAN | MAPK3 | ENSG00000102882 | Mitogen-activated protein kinase 3 |
| MK01_HUMAN | MAPK1 | ENSG00000100030 | Mitogen-activated protein kinase 1 |
| GNAI2_HUMAN | GNAI2 | ENSG00000114353 | Guanine nucleotide-binding protein G(i) subunit alpha-2 |
| GNAS_HUMAN | GNAS | ENSG00000087460 | Guanine nucleotide-binding protein G(s) subunit alpha isoforms short |
| GCR_HUMAN | NR3C1 | ENSG00000113580 | Glucocorticoid receptor |
| IL1B_HUMAN | IL1B | ENSG00000125538 | Interleukin-1 beta |
| IL6_HUMAN | IL6 | ENSG00000136244 | Interleukin-6 |
| IL8_HUMAN | IL8 | ENSG00000169429 | Interleukin-8 |
| I23O1_HUMAN | IDO1 | ENSG00000131203 | "Indoleamine 2,3-dioxygenase 1" |
| MK14_HUMAN | MAPK14 | ENSG00000112062 | Mitogen-activated protein kinase 14 |
| MK08_HUMAN | MAPK8 | ENSG00000107643 | Mitogen-activated protein kinase 8 |
| DUS1_HUMAN | DUSP1 | ENSG00000120129 | Dual specificity protein phosphatase 1 |
| MCR_HUMAN | NR3C2 | ENSG00000151623 | Mineralocorticoid receptor |
| DCOR_HUMAN | ODC1 | ENSG00000115758 | Ornithine decarboxylase |
| P2RX7_HUMAN | P2RX7 | ENSG00000089041 | P2X purinoceptor 7 |
| TSPOA_HUMAN | TSPO | ENSG00000100300 | Translocator protein |
| PPCE_HUMAN | PREP | ENSG00000085377 | Prolyl endopeptidase |
| RGS2_HUMAN | RGS2 | ENSG00000116741 | Regulator of G-protein signaling 2 |
| SC6A4_HUMAN | SC6A4 | ENSG00000108576 | Sodium-dependent serotonin transporter |
| S10AA_HUMAN | S10AA | ENSG00000197747 | Protein S100-A10 |
| VMAT2_HUMAN | SLC18A2 | ENSG00000165646 | Synaptic vesicular amine transporter |
