# Supplementary material for: Gene Expression Profiles in Relation to Tension and Dissociation in Borderline Personality Disorder
Source: PLoS One. 2013 Aug 12;8(8):e70787. doi: 10.1371/journal.pone.0070787 (PMC3741306; doi:10.1371/journal.pone.0070787)
Supplement: Table S3 — Targets and primers. (DOCX) [file pone.0070787.s003.docx]

**Table S3: Targets and primers**

| Target |  | Primer sequence |
| --- | --- | --- |
| adenosine deaminase | ADA | F = GGTGGTGGAGCTGTGTAAGAAGTAC |
|  |  | R = CTTCCTGGGATGGTCTCATCTC |
|  |  | P = CAGCAGACCGTGGTAGCCATTGACCT |
| beta-arrestin 1 | ARRB1 | F = AGACACGAACTTGGCCTCTAGC |
|  |  | R = TTGTAGGAAACAATGATCCCCAG |
|  |  | P = TTGAGGGAAGGTGCCAACCGTGAGAT |
| beta-arrestin 2 | ARRB2 | F = TCTTCCATGCTCCGTCACAC |
|  |  | R = CGAATCTCAAAGTCTACGCCG |
|  |  | P = AGCCAGGCCCAGAGGATACAGGAAA |
| CD8 alpha | CD8A | F = TTCCGCCGAGAGAACGAG |
|  |  | R = AAGACCGGCACGAAGTGG |
|  |  | P = TCGGCCCTGAGCAACTCCATCATGTA |
| CD8 beta | CD8B | F = TGACAGTCACCACGAGTTCCTG |
|  |  | R = TCTCCTGTTCCACCTCTTCACC |
|  |  | P = CTCTGGGATTCCGCAAAAGGGACTAT |
| cAMP responsive element binding protein 1 | CREB1 | F = CTGGCTAACAATGGTACCGATG |
|  |  | R = GTGGTCTGTGCATACTGTAGAATGG |
|  |  | P = CATGACCAATGCAGCAGCCACTCA |
| cAMP responsive element binding protein 2 | CREB2 | F = CACGTTGGATGACACTTGTGATC |
|  |  | R = CTGGGAGATGGCCAATTGG |
|  |  | P = ACTAATAAGCAGCCCCCCCAGACGGT |
| dipeptidyl peptidase IV | DPP4 | F = GTGTCATTCAGTAAAGAGGCGAAG |
|  |  | R = CTCAGCCCTTTATCATTCACGC |
|  |  | P = TTCCGGTCCTGGTCTGCCCCTCTATA |
| extracellular signal-regulated kinase 1 | ERK1 | F = TGACGGAGTATGTGGCTACGC |
|  |  | R = CCACAGACCAGATGTCGATGG |
|  |  | P = CTGGTACCGGGCCCCAGAGATCAT |
| extracellular signal-regulated kinase 2 | ERK2 | F = TAACGTTCTGCACCGTGACC |
|  |  | R = CAGGCCAAAGTCACAGATCTTG |
|  |  | P = ACCTGCTGCTCAACACCACCTGTGAT |
| guanine nucleotide binding protein alpha i2 | GNAI2 | F = AGGCGTGCTCCCTGATGAC |
|  |  | R = GCTCCAGGTCGTTCAGGTAGTAG |
|  |  | P = AGGCCTGCTTTGGCCGCTCAA |
| guanine nucleotide binding protein alpha s (long) | GNAS | F = GACTATGTGCCGAGCGATCAG |
|  |  | R = GTCCACCTGGAACTTGGTCTCA |
|  |  | P = CTGCTTCGCTGCCGTGTCCTGA |
|  |  |  |
| alpha-glucocorticoid receptor | NR3C1 | F = TCCCTGGTCGAACAGTTTTTTC |
|  |  | R = TTTGGGAGGTGGTCCTGTTG |
|  |  | P = TGTAAGCTCTCCTCCATCCAGCTCCTCAA |
| interleukin 1, beta | IL1B |  |
|  |  | F = GATGGCCCTAAACAGATGAAGTG |
|  |  | R = CCTGAAGCCCTTGCTGTAGTG |
|  |  | P = ATGGCGGCATCCAGCTACGAATCTC |
| interleukin 6 | IL6 |  |
|  |  | F = AGCCACTCACCTCTTCAGAACG |
|  |  | R = CATGTCTCCTTTCTCAGGGCTG |
|  |  | P = CAAATTCGGTACATCCTCGACGGCAT |
| interleukin 8 | IL8 |  |
|  |  | F = CTGCTAGCCAGGATCCACAAG |
|  |  | R = CTGTGAGGTAAGATGGTGGCTAATAC |
|  |  | P = CTTGTTCCACTGTGCCTTGGTTTCTCCTT |
| indoleamine-pyrrole 2,3 dioxygenase | IDO1 |  |
|  |  | F = GCTTCGAGAAAGAGTTGAGAAGTTAAAC |
|  |  | R = GACCTTTGCCCCACACATATG |
|  |  | P = CTCACAGACCACAAGTCACAGCGCCTT |
| p38 mitogen activated protein kinase 14 | MAPK14 |  |
|  |  | F = CGGCAGGAGCTGAACAAGAC |
|  |  | R = AGCAGCACACACAGAGCCATAG |
|  |  | P = CCGAGCGTTACCAGAACCTGTCTCCA |
| mitogen-activated protein kinase 8 | MAPK8 |  |
|  |  | F = CCAACACCCGTACATCAATGTC |
|  |  | R = CACTCTTCTATTGTGTGTTCCCTTTC |
|  |  | P = CACCACCAAAGATCCCTGACAAGCAGTT |
| map kinase phosphatase 1 | DUSP1 |  |
|  |  | F = GCCAGGCAGGCATTTCC |
|  |  | R = ATGCTTCGCCTCTGCTTCAC |
|  |  | P = TCAGCCACCATCTGCCTTGCTTACCTT |
| mineralocorticoid receptor | NR3C2 |  |
|  |  | F = AGCCCAGAGGAAGGGACAAC |
|  |  | R = TGTGAGCGCTCGTGAGATTG |
|  |  | P = CTCCTGCAAAAGAACCCTCGGTCAACA |
| ornithine decarboxylase 1 | ODC1 |  |
|  |  | F = CCATGTAGGAAGCGGCTGTAC |
|  |  | R = TCAGCCCCCATGTCAAAAAC |
|  |  | P = ATCCTGAGACCTTCGTGCAGGCAATCT |
| purinergic receptor P2X7 | P2RX7 |  |
|  |  | F = GCTGTCGCTCCCATATTTATCC |
|  |  | R = CACAATGGACTCGCACTTCTTC |
|  |  | P = CTGTCAGCCCTGTGTGGTCAACGAATAC |
| benzodiazapine receptor (peripheral-type) | TSPO |  |
|  |  | F = CTGGTCTGGAAAGAGCTGGG |
|  |  | R = CAGCAGGAGATCCACCAAGG |
|  |  | P = CCCCATCTTCTTTGGTGCCCGAC |
| prolyl endopeptidase | PREP |  |
|  |  | F = GGGAATATGACTACGTGACCAATG |
|  |  | R = GGATCCCTGAAGTCAATGTTGATC |
|  |  | P = CATTCAAGACGAATCGCCAGTCTCCC |
| regulator of G-protein signaling 2 | RGS2 |  |
|  |  | F = GATTGGAAGACCCGTTTGAGC |
|  |  | R = CAGGAGAAGGCTTGATGAAAGC |
|  |  | P = CTGGGAAGCCCAAAACCGGCAA |
| S100 calcium binding protein A10 (p11) | S100A10 |  |
|  |  | F = AGGAGTTCCCTGGATTTTTGG |
|  |  | R = GCCCACTTTGCCATCTCTACAC |
|  |  | P = CAAAAAGACCCTCTGGCTGTGGACAAAA |
| serotonin transporter | SLC6A4 |  |
|  |  | F = CATGGCTGAGATGAGGAATGAAG |
|  |  | R = GCTGGCATGTTGGCTATCG |
|  |  | P =ACGCAGGTCCCAGCCTCCTCTTCAT |
| vesicle monoamine transporter 2 | SLC18A2 |  |
|  |  | F = TGGATTCGTCAATGATGCCTATC |
|  |  | R = ATGCCACATCCGCAATGG |
|  |  | P = AGACCTGCGGCACGTGTCCGTCTA |
| beta-2-microglobulin | B2M | F= CATCCAGCAGAGAATGGAAAGTC |
|  |  | R= TTCTCTCTCCATTCTTCAGTAAGTCAAC |
|  |  | P= TGTGTCTGGGTTTCATCCATCCGACATT |
| glyceraldehyde-3-phosphate dehydrogenase | GAPDH | F= CTGCCCCCTCTGCTGATG |
|  |  | R= GCTGATGATCTTGAGGCTGTTG |
|  |  | P= TTCGTCATGGGTGTGAACCATGAGAAGT |
| peptidylpropyl isomerase A (cyclophilin A) | PPIA | F= TCAAACTGAAGCACTACGGGC |
|  |  | R= CATGCTTGCCATCTAGCCAG |
|  |  | P= AAAGACACCAACGGCTCCCAGTTCTTCA |
| ribosomal protein, large, P0 | RPLP0 | F= GAGTGATGTGCAGCTGATCAAGAC |
|  |  | R= ATGACCAGCCCAAAGGAGAAG |
|  |  | P= AAGCCACGCTGCTGAACATGCTCAA |
| ribosomal protein L13a | RPL13A | F= CCTGGAGGAGAAGAGGAAAGAGA |
|  |  | R= TTGAGGACCTCTGTGTATTTGTCAA |
|  |  | P= CCACTACCGGAAGAAGAAACAGCTCATGAG |
| TATA box binding protein (transcription factor IID) | TBP | F= GCTGCGGTAATCATGAGGATAAG |
|  |  | R= CTCCTGTGCACACCATTTTCC |
|  |  | P= AGCCACGAACCACGGCACTGATTTT |
| ubiquitin C | UBC | F= GATTTGGGTCGCAGTTCTTG |
|  |  | R= TGCCTTGACATTCTCGATGGT |
|  |  | P= ATCGCTGTGATCGTCACTTGACAAT |
| ^†^ F = Forward primer; R=Reverse primer; P = Probe ; all sequences shown in 5' to 3' orientation | | |
