# Supplementary material for: Gene Expression Profiles in Relation to Tension and Dissociation in Borderline Personality Disorder
Source: PLoS One. 2013 Aug 12;8(8):e70787. doi: 10.1371/journal.pone.0070787 (PMC3741306; doi:10.1371/journal.pone.0070787)
Supplement: Table S4 — Normalization transcripts. (DOCX) [file pone.0070787.s004.docx]

**Table S 4: Normalization transcripts**

| Gene Name | Abbreviation | Gene Accession Number |
| --- | --- | --- |
| beta-2-microglobulin | B2M | NM_004048 |
| glyceraldehyde-3-phosphate dehydrogenase | GAPDH | NM_002046 |
| peptidylpropyl isomerase A (cyclophilin A) | PPIA | NM_021130 |
| ribosomal protein, large, P0 | RPLP0 | NM_001002 |
| ribosomal protein L13a | RPL13A | NM_012423 |
| TATA box binding protein (transcription factor IID) | TBP | NM_003194 (M34960) |
| ubiquitin C | UBC | NM_021009 (M26880) |
