# Supplementary material for: Gene Expression Profiles in Relation to Tension and Dissociation in Borderline Personality Disorder
Source: PLoS One. 2013 Aug 12;8(8):e70787. doi: 10.1371/journal.pone.0070787 (PMC3741306; doi:10.1371/journal.pone.0070787)
Supplement: Table S5 — Gene expression level associated with BSL score sorted by FDR. (DOCX) [file pone.0070787.s005.docx]

**Table S 5: Gene expression level associated with BSL score sorted by FDR.**

| Gene | Estimated regression coefficients | FDR | p-value |
| --- | --- | --- | --- |
| TSPO | 0.2444 | 0.78049 | 0.0269 |
| NR3C1 | -0.09691 | 0.95268 | 0.1405 |
| MAPK1 | -0.1015 | 0.95268 | 0.1551 |
| CREB1 | -0.09522 | 0.95268 | 0.1617 |
| DUSP1 | -0.1448 | 0.95268 | 0.1889 |
| MAPK8 | -0.07677 | 0.95268 | 0.2265 |
| IL1B | -0.1081 | 0.95268 | 0.2768 |
| ARRB2 | -0.09824 | 0.95268 | 0.3037 |
| S100A10 | 0.04363 | 0.95268 | 0.3102 |
| ADA | 0.1086 | 0.95268 | 0.3285 |
| ARRB1 | -0.05974 | 0.95832 | 0.3780 |
| GNAS | -0.06068 | 0.95832 | 0.4409 |
| GNAI2 | -0.04989 | 0.95832 | 0.4511 |
| RGS2 | -0.06544 | 0.95832 | 0.5051 |
| ODC1 | -0.06127 | 0.95832 | 0.5683 |
| MAPK3 | -0.04138 | 0.95832 | 0.5812 |
| SLC6A4 | -0.07051 | 0.95832 | 0.5904 |
| DPP4 | 0.03364 | 0.95832 | 0.6316 |
| NR3C2 | -0.03897 | 0.95832 | 0.6902 |
| SLC18A2 | 0.03309 | 0.95832 | 0.6908 |
| CD8A | 0.04695 | 0.95832 | 0.7071 |
| PREP | -0.01986 | 0.95832 | 0.7311 |
| IL6 | -0.02437 | 0.95832 | 0.7601 |
| ATF2 | 0.01241 | 0.97916 | 0.8205 |
| IDO1 | 0.02250 | 0.97916 | 0.8879 |
| CD8B | 0.008773 | 0.97916 | 0.9163 |
| MAPK14 | -0.00842 | 0.97916 | 0.9172 |
| P2RX7 | -0.00476 | 0.97916 | 0.9454 |
| IL8 | 0.000956 | 0.99392 | 0.9939 |
