# Supplementary material for: Gene Expression Profiles in Relation to Tension and Dissociation in Borderline Personality Disorder
Source: PLoS One. 2013 Aug 12;8(8):e70787. doi: 10.1371/journal.pone.0070787 (PMC3741306; doi:10.1371/journal.pone.0070787)
Supplement: Table S6 — Gene expression level associated with Tension/Non-tension state sorted by FDR. (DOCX) [file pone.0070787.s006.docx]

**Table S 6: Gene expression level associated with Tension/Non-tension state sorted by FDR.**

| Gene | Estimated regression coefficients | FDR | p-value |
| --- | --- | --- | --- |
| IL6 | -0.1546 | 0.48816 | 0.0368 |
| MAPK3 | 0.1223 | 0.48816 | 0.0504 |
| GNAS | 0.1477 | 0.48816 | 0.0505 |
| ARRB2 | 0.1425 | 0.57196 | 0.0826 |
| ARRB1 | 0.1003 | 0.57196 | 0.1484 |
| SLC18A2 | 0.1091 | 0.57196 | 0.1533 |
| PREP | 0.07736 | 0.57196 | 0.1550 |
| IL1B | 0.1179 | 0.57196 | 0.1645 |
| SLC6A4 | 0.1524 | 0.57196 | 0.1836 |
| S100A10 | 0.05653 | 0.57196 | 0.2479 |
| DPP4 | 0.05688 | 0.57196 | 0.2683 |
| CD8A | -0.1029 | 0.57196 | 0.2788 |
| RGS2 | 0.07863 | 0.57196 | 0.2962 |
| ATF2 | -0.03951 | 0.57196 | 0.3326 |
| NR3C2 | 0.08863 | 0.57196 | 0.3430 |
| ADA | -0.09312 | 0.57196 | 0.3515 |
| ODC1 | 0.07558 | 0.57196 | 0.3547 |
| GNAI2 | 0.07322 | 0.57196 | 0.3550 |
| MAPK1 | 0.04914 | 0.65504 | 0.4292 |
| P2RX7 | 0.04615 | 0.75632 | 0.5445 |
| NR3C1 | 0.03976 | 0.75632 | 0.5538 |
| CREB1 | 0.03888 | 0.75632 | 0.5738 |
| IL8 | -0.04487 | 0.79080 | 0.6272 |
| DUSP1 | 0.03311 | 0.87266 | 0.7222 |
| MAPK14 | 0.01948 | 0.90115 | 0.7769 |
| TSPO | 0.02096 | 0.90707 | 0.8665 |
| MAPK8 | 0.01079 | 0.90707 | 0.8727 |
| CD8B | -0.00807 | 0.90707 | 0.8758 |
| IDO1 | 0.01082 | 0.91408 | 0.9141 |
