# Supplementary material for: Gene Expression Profiles in Relation to Tension and Dissociation in Borderline Personality Disorder
Source: PLoS One. 2013 Aug 12;8(8):e70787. doi: 10.1371/journal.pone.0070787 (PMC3741306; doi:10.1371/journal.pone.0070787)
Supplement: Table S7 — Gene expression level associated with DSS tension item sorted by FDR. (DOCX) [file pone.0070787.s007.docx]

**Table S 7: Gene expression level associated with DSS tension item sorted by FDR.**

| **Gene** | **Estimated regression coefficients** | **FDR** | **p-value** |
| --- | --- | --- | --- |
| IL6 | 0.02398 | 0.99743 | 0.0795 |
| S100A10 | -0.01434 | 0.99743 | 0.1027 |
| ATF2 | 0.007772 | 0.99743 | 0.1934 |
| PREP | -0.00964 | 0.99743 | 0.3178 |
| DUSP1 | 0.01556 | 0.99743 | 0.3628 |
| NR3C2 | -0.01543 | 0.99743 | 0.3797 |
| ARRB1 | -0.01035 | 0.99743 | 0.4081 |
| GNAS | -0.01172 | 0.99743 | 0.4091 |
| MAPK3 | -0.00829 | 0.99743 | 0.4330 |
| ARRB2 | -0.01160 | 0.99743 | 0.4401 |
| IL8 | 0.01224 | 0.99743 | 0.4721 |
| ADA | 0.01382 | 0.99743 | 0.4745 |
| DPP4 | -0.00582 | 0.99743 | 0.5327 |
| CD8A | 0.009766 | 0.99743 | 0.6042 |
| MAPK1 | 0.005212 | 0.99743 | 0.6393 |
| IDO1 | -0.00754 | 0.99743 | 0.6493 |
| MAPK8 | -0.00430 | 0.99743 | 0.7104 |
| CREB1 | -0.00449 | 0.99743 | 0.7218 |
| SLC6A4 | -0.00629 | 0.99743 | 0.7657 |
| CD8B | -0.00301 | 0.99743 | 0.7677 |
| MAPK14 | 0.003292 | 0.99743 | 0.7845 |
| SLC18A2 | -0.00375 | 0.99743 | 0.7845 |
| ODC1 | -0.00407 | 0.99743 | 0.8027 |
| P2RX7 | -0.00208 | 0.99743 | 0.8843 |
| TSPO | 0.003065 | 0.99743 | 0.8945 |
| GNAI2 | -0.00189 | 0.99743 | 0.8961 |
| IL1B | -0.00022 | 0.99743 | 0.9887 |
| RGS2 | 0.000180 | 0.99743 | 0.9895 |
| NR3C1 | 0.000038 | 0.99743 | 0.9974 |
